# Memristors with Biomaterials for Biorealistic Neuromorphic Applications

**DOI:** 10.1002/smsc.202200028

**Published:** 2022-08-22

**Authors:** Jiaqi Xu, Xiaoning Zhao, Xiaoli Zhao, Zhongqiang Wang, Qingxin Tang, Haiyang Xu, Yichun Liu

**Affiliations:** ^1^ Key Laboratory of UV Light-Emitting Materials and Technology of Ministry of Education Northeast Normal University Changchun 130024 China

**Keywords:** biomaterials, biorealistic applications, green electronics, memristors, resistive switching

## Abstract

Electronic devices with biomaterials have paved a way toward “green electronics” to create a sustainable future. Memristors are drawing growing attention with integrated sensing, memory, and computing for future artificial intelligence applications. Biomaterial is an emerging class of memristive materials (the device is called as biomemristor) for transient and/or biodegradable purpose. Importantly, several unique features such as faithful synaptic behaviors, bimodal switching, and biovoltage operations are observed in biomemristors. Moreover, the biomemristors are suitable for human‐related applications due to the inherent biocompatibility of biomaterials and flexibility of the device with ultrathin thickness. These features make the biomemristors promising for biorealistic neuromorphic applications. Herein, the state of the art of biomemristors are comprehensively summarized and systematically discussed with particular attention on their unique biorealistic features. Challenges and prospects toward the further development of biomemristors are also provided and discussed.

## Graphical abstract

As emerging memristive materials, biomaterials hold great promise for “green” electronics to create a sustainable future with unique biodegradable and biocompatible properties. This review systematically outlines the state‐of‐the‐art development of biomemristor devices with a focus on their unique features for biorealistic neuromorphic applications. Future key challenges and opportunities are suggested.

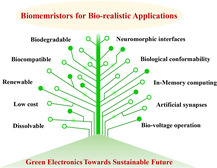

## 1 Introduction

There have been numerous efforts and significant advancements in modern electronic technology over the past 100 years. Electronic components like memory, sensor, light‐emitting diodes, battery, etc. are indispensable in human daily life. The production of electronic components is growing exponentially toward the era of the Internet of Things (IoT), artificial intelligence, and fifth‐generation mobile networks. The lifetime of electronic products is shortened. Along with that, a large amount of electronic waste (e‐waste) is generated. According to statistics,^[^ ^1^ ^]^ global e‐waste will grow by 21% in 5 years but recycling cannot keep up with the growing rate. E‐waste with hazardous and/or toxic chemicals is exposed to the environment every day and now makes up 5% of all solid waste.^[^ ^2^, ^3^ ^]^ To address these issues, on a device level, growing interest in “green electronics” where devices are made of renewable and sustainable materials is developed.^[^ ^4^, ^5^, ^6^, ^7^, ^8^, ^9^, ^10^ ^]^ According to European standards, more than 90% of materials used must be degraded harmlessly within 6 months after abandonment for “green” electronic devices.^[^ ^11^ ^]^ Natural abundant biomaterials are the best candidates because they are renewable, biocompatible, and biodegradable. Over the past few years, numerous efforts have been devoted to develop “green” electronic devices involving transistors, diodes, capacitors, and resistors with biomaterials.^[^ ^12^, ^13^, ^14^, ^15^, ^16^, ^17^, ^18^ ^]^ Biomaterials not only can address the e‐waste issue, but also have the advantages of dissolvability, flexibility, and biocompatibility, and they can be widely used in secrecy storage, wearable electronics, biomedicals, and so on.

On the other hand, traditional von Neumann architecture that operated based on the physical separation between processor and memory is facing challenges in speed and energy (which is known as von Neumann bottleneck).^[^ ^19^, ^20^ ^]^ Efforts are devoted to search for novel devices to overcome the von Neumann bottleneck. Among the candidates, a memristor, which was proposed by Chua^[^ ^21^ ^]^ in 1971 and physically developed by Hewlett Packard labs^[^ ^22^ ^]^ in 2008, has been regarded as the most promising technology for integrated sensing, memory, and computing applications.^[^ ^23^, ^24^, ^25^, ^26^, ^27^, ^28^ ^]^ In 2014, Leon Chua further pushed the idea of memristor device as any two‐terminal device that exhibits a pinched hysteresis loop in the current–voltage characteristics.^[^ ^29^ ^]^ Such a definition greatly extends the scope of memristors. Memristor devices can be found in simple structure with excellent miniaturization potential to tens of nanometers. The device conductance can be modulated discretely or continuously (which is also termed resistive switching (RS) behavior) by applying a voltage bias.^[^ ^30^ ^]^ RS is also at the base of the behavior of the memristor devices. One important application of memristors is the resistive random‐access memory which is often referred to as a memristor.^[^ ^31^ ^]^ Memristor can be operated with fast speed within subnanoseconds and low energy consumption of femtojoule.^[^ ^32^, ^33^, ^34^, ^35^, ^36^ ^]^ These features make it good candidates for broad applications of nonvolatile memory, computing, and neuromorphic applications. Recent research has also explored the use of light and humidity to control or affect the memristive behavior of the device, which expands the application scope of memristors to integrated sensing, memory, and computing functions.^[^ ^37^, ^38^, ^39^, ^40^, ^41^, ^42^ ^]^

**Figure 1:**
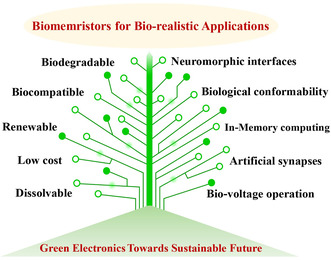
The characteristics of biomaterials and some representative biorealistic features of biomemristors.

To date, a variety of materials including transition metal oxides, organics, biomaterials, chalcogenides, carbon‐based materials, polymers, etc. have been explored as building blocks for memristor devices.^[^ ^43^, ^44^, ^45^, ^46^, ^47^, ^48^ ^]^ Biomaterials with advantages of biodegradability, biocompatibility, renewable nature, low‐cost, and water‐dissolvable characteristics could be a complement to memristive material systems (**Figure** **1**).^[^ ^49^, ^50^, ^51^, ^52^, ^53^, ^54^, ^55^ ^]^ Biomaterials can be produced sustainably and modified with diverse functions than traditional electronic materials. Benefiting from the synapse‐analogous, humidity‐sensitive, and metallization‐catalytic characteristics of some biomaterials and biomemristor devices, unique features such as faithful synaptic behaviors, bimodal switching, and biovoltage operation have been observed. Moreover, the biomemristors are suitable for human‐related applications due to the inherent biocompatibility of biomaterials and mechanical flexibility of the device with ultrathin thickness.^[^ ^56^ ^]^ These features make the biomemristor promising for biorealistic neuromorphic applications. In this work, recent advances in the development of biomemristor devices are reviewed. We begin with a brief introduction of biomemristors, along with their device configuration, switching characteristics, and working mechanisms. In the following, unique features of biomemristor devices with biorealistic functions are highlighted. Finally, challenges and perspectives of biomemristors for future neuromorphic applications are provided and discussed.

## 2 Biomemristors: Materials, Devices, Switching Characteristics, and Mechanisms

It is well known that memristors can be classified into digital or analog type depending on whether the RS behavior is discrete or continuous, respectively.^[^ ^57^ ^]^ Digital‐type memristors with binary RS can be developed as memory. While analog‐type memristors with continuously tunable resistance states are primarily suitable for neuromorphic applications, in the early stages of biomemristors, work mainly focuses on studying the binary RS phenomenon and their related physical mechanisms. Subsequently, biomemristors with analog RS behaviors have been successively developed for neuromorphic applications. These two types of memristors are similar in terms of device structure, materials, and switching mechanisms, to a certain extent. To make the paper more comprehensive and adequate, a description on the materials, devices, and working mechanisms is given, regardless of whether it is digital‐ or analog‐type RS.

**Figure 2:**
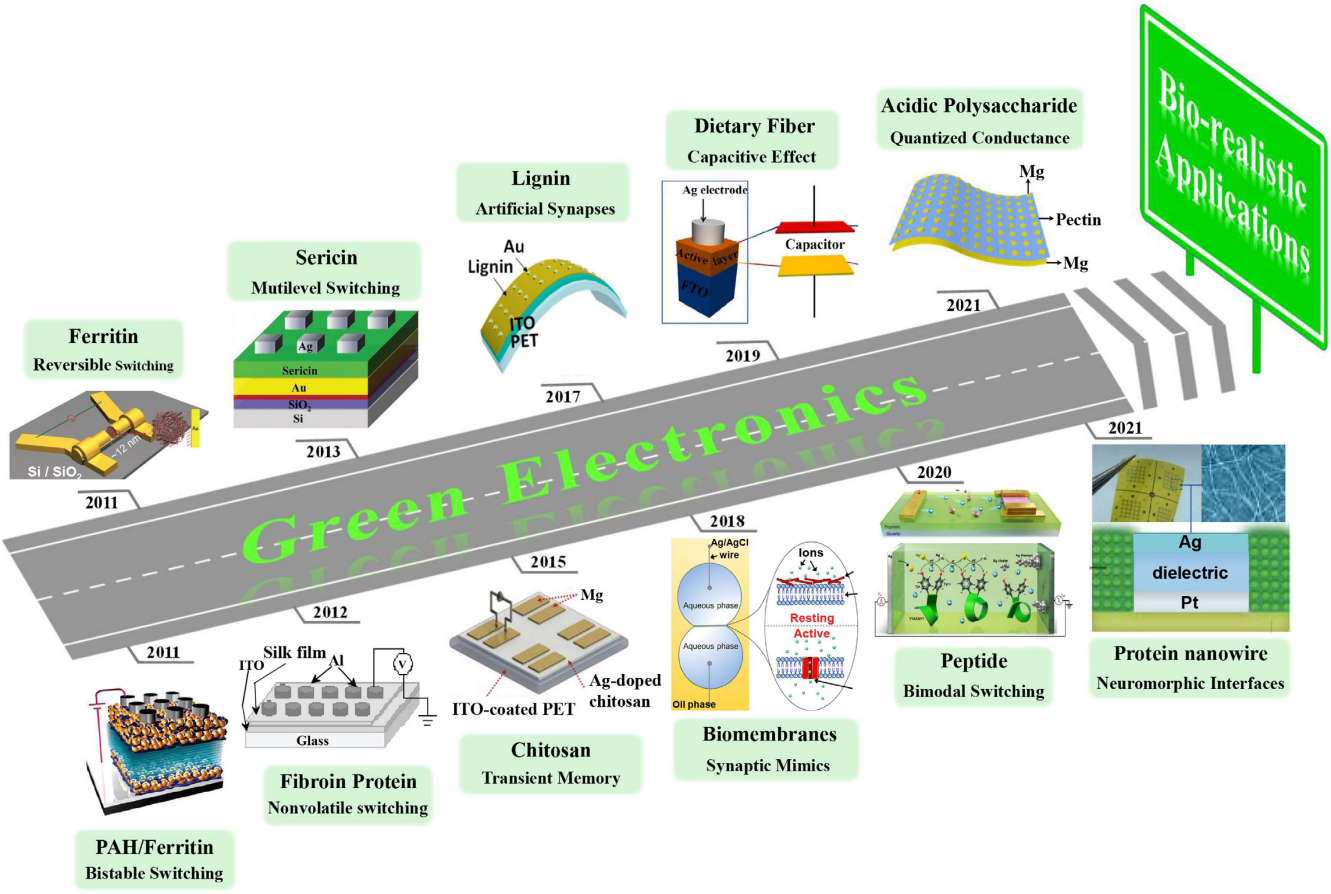
Some representive works in the development of biomemristors. Ferritin: Reproduced with permission.^[^ ^58^ ^]^ Copyright 2011, Wiley‐VCH. PAH/Ferritin: Reproduced with permission.^[^ ^59^ ^]^ Copyright 2011, American Chemical Society. Fibroin Protein: Reproduced with permission.^[^ ^60^ ^]^ Copyright 2012, Wiley‐VCH. Sericin: Reproduced with permission.^[^ ^61^ ^]^ Copyright 2013, Wiley‐VCH. Chitosan: Reproduced with permission.^[^ ^62^ ^]^ Copyright 2015, Wiley‐VCH. Lignin: Reproduced with permission.^[^ ^63^ ^]^ Copyright 2017, American Chemical Society. Biomembranes: Reproduced with permission.^[^ ^64^ ^]^ Copyright 2018, American Chemical Society. Dietary Fiber: Reproduced with permission.^[^ ^65^ ^]^ Copyright 2019, Elsevier Ltd. Peptide: Reproduced under the terms of the CC‐BY 4.0 license.^[^ ^66^ ^]^ Copyright 2020, The Authors, published by Springer Nature. Acidic Polysaccharide: Reproduced with permission.^[^ ^67^ ^]^ Copyright 2021, Wiley‐VCH. Protein nanowire: Reproduced under the terms of the CC‐BY 4.0 license.^[^ ^68^ ^]^ Copyright 2021, The Authors, published by Springer Nature.

Biomaterials are defined as materials formed during the growth cycles of all living organisms in nature. Biomaterials are sustainable and renewable because they can be extracted from organisms. In the past decade, natural biomaterials have received great attention in the fabrication of electronic devices. Biomaterials have also been successfully used as active layers of memristors, including, but not limited to, biomemrbranes, proteins and their composites, saccharides, and deoxyribonucleic acid (DNA) (**Figure** **2**). Some plant materials (wood, leaves, fruit, flowers) were also developed for memristors. In the following sections, we will review the works on biomemristors with a focus mainly on materials, devices, switching characteristics, and their working mechanisms.

**Figure 3:**
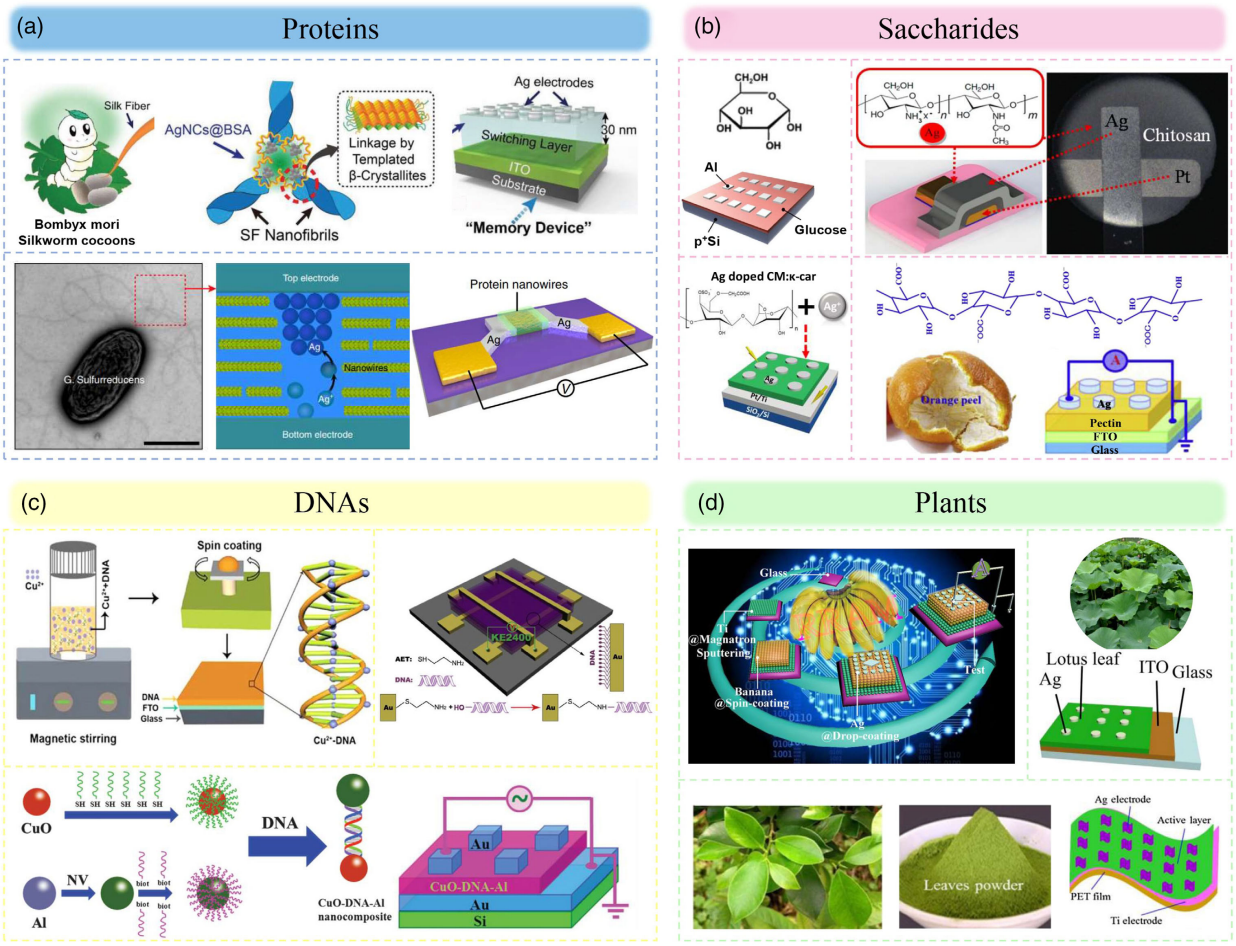
Biomaterials used for memristors. a) Proteins. Top: Reproduced with permission.^[^ ^71^ ^]^ Copyright 2019, Wiley‐VCH. Bottom: Reproduced under the terms of the CC‐BY 4.0 license.^[^ ^83^ ^]^ Copyright 2020, The Authors, published by Springer Nature. b) Saccharides. Top left: Reproduced with permission.^[^ ^84^ ^]^ Copyright 2018, Wiley‐VCH. Top right: Reproduced with permission.^[^ ^85^ ^]^ Copyright 2015, American Chemical Society. Bottom left: Reproduced with permission.^[^ ^86^ ^]^ Copyright 2018, American Chemical Society. Bottom right: Reproduced with permission.^[^ ^87^ ^]^ Copyright 2017, Elsevier Ltd. c) DNAs. Bottom: Reproduced with permission.^[^ ^88^ ^]^ Copyright 2019, Royal Society of Chemistry. Top left: Reproduced with permission.^[^ ^89^ ^]^ Copyright 2015, IOP Publishing. Top right: Reproduced with permission.^[^ ^90^ ^]^ Copyright 2015, Elsevier. d) Plants. Top left: Reproduced with permission.^[^ ^91^ ^]^ Copyright 2018, American Chemical Society. Top right: Reproduced with permission.^[^ ^92^ ^]^ Copyright 2018, Elsevier Ltd. Bottom: Reproduced with permission.^[^ ^93^ ^]^ Copyright 2017, Elsevier Ltd.

Proteins are known to be the most abundant macromolecules in living organisms to perform a wide range of biological functions.^[^ ^69^, ^70^ ^]^ Proteins are dispersible, soluble, biocompatible, and biodegradable; they can be synthesized biotechnologically and chemically. Proteins such as ferritin, fibroin, keratin, et al. have been developed for biomemristor devices (**Figure** **3**a).^[^ ^71^, ^72^, ^73^ ^]^ Though biomemristors with proteins showed remarkable switching characteristics, most of the proteins involve complicated extraction or purification processes, which inevitably add complexity to the device fabrication process. Proteins obtained directly from fresh eggs,^[^ ^74^, ^75^, ^76^, ^77^ ^]^ *Bombyx mori*,^[^ ^60^, ^78^, ^79^, ^80^, ^81^ ^]^ spider,^[^ ^82^ ^]^ and bacterium *Geobacter sulfurreducens* ^[^ ^68^, ^83^ ^]^ without additional chemical processes have also been investigated intensively as a promising electronic material for biomemristors.

Saccharides widely exist in animals, plants, and microorganisms. Most saccharides are nontoxic, and they have been used extensively in food products, cosmetics, and pharmaceutical applications.^[^ ^94^ ^]^ In the presence of hydroxyl and/or the carboxyl groups, polysaccharides have a strong affinity to water molecules and strong interaction among saccharide molecules via hydrogen bonding.^[^ ^95^ ^]^ Saccharides are suitable materials in thin‐film preparation due to their excellent processability. These features make it a promising electronic material for transient and implantable applications.^[^ ^62^, ^96^ ^]^ In these years, saccharides such as glucose,^[^ ^84^ ^]^ chitosan,^[^ ^85^ ^]^ carrageenan,^[^ ^86^ ^]^ pectin,^[^ ^87^ ^]^ et al. have been developed for biomemristors (Figure 3b). An advantage of saccharides is the simple synthesis method. For example, Sun et al.^[^ ^87^ ^]^ reported the pectin‐based memristor with a simple microwave‐assisted extraction method for the first time.

DNA is the hereditary material in humans and almost all other organisms. The DNA molecule consists of two strands that wind around one another with a helix structure.^[^ ^88^ ^]^ DNA has been reported as a good template for metal nanoparticle synthesis for various applications.^[^ ^89^, ^90^ ^]^ Metal ions can stabilize the DNA structure by coordinating with the phosphodiester backbone. Therefore, DNA can behave as a host for metal ions and DNA–metal composites have been proposed as promising building blocks for biomemristors (Figure 3c).

In addition, the variety of natural available plants also provides an abundance of unexplored sources for researchers. Plant organisms, including wood, leaves, fruit, and flowers, are naturally abundant, sustainable, biocompatible, and biodegradable. These materials do not require complex chemical syntheses that result in environmental problems and energy consumption. In recent years, driven by the demand for novel green and smart applications, these natural plants have also been proposed as active building blocks for biomemristors (Figure 3d).^[^ ^63^, ^65^, ^91^, ^92^, ^93^, ^97^, ^98^, ^99^, ^100^ ^]^

A detailed summary of basic RS parameters and working mechanisms from some representative works on biomamristors is provided in **Table** **1**. Though outstanding performances have been reported in some biomemristor devices such as fast switching speed, high switching uniformity, and low power consumption, it is still a challenge to combine all the superior characteristics in a single device. There is still a large performance gap between biomemristor devices and inorganic counterparts.^[^ ^101^, ^102^, ^103^, ^104^ ^]^

**Table 1:** Summary of the switching performances of some reported biomemristor devices

Materials	Device structure	Mechanism	OFF/ON ratio	Cyclic number	Retention [s]	References
Proteins	Au/ferritin/Au	Valence state alternation	<10	10	/	[58]
	Ag/(PAH/ferritin)n/Pt	Valence state alternation	10^3^	300	10^4^	[59]
	Ag/sericin/Au	Charge trap/detrap	10^6^	21	10^3^	[61]
	Al/gelatin/ITO	Carbon filament	10^6^	120	10^5^	[105]
	Ag/fibroin/Au	Ag filament	>10^7^	20	10^4^	[72]
	Mg/egg albumen/W	Mg filament	10^3^	120	10^4^	[76]
	Mg/fibroin/Mg	Mg filament	10^3^	50	10^4^	[106]
	Cu/rDnaJ/Pt	Cu filament	10^6^	100	10^6^	[107]
	Al/CDs‐silk protein/ITO	Charge trap/detrap	10^6^	100	10^6^	[108]
	Ag/AgNCs@BSA/ITO	Ag filament	10^3^	100	10^3^	[71]
	Au/silk‐Ag nanowires/Au	Ag filament	10^6^	/	10^−3^ to 10^4^	[109]
	Ag/protein nanowires/Pt	Ag filament	10^6^	10^4^	/	[83]
	Ag/protein nanowires/Pt	Ag filament	/	10^4^	/	[68]
	Ag/fibroin‐AgNCs/ITO	Ag filament	10^7^	300	10^4^	[78]
Saccharides	Mg/Ag‐doped chitosan/Mg	Metallic filament	10^2^	60	10^4^	[62]
	Ag/Ag‐doped chitosan/Pt	Ag filament	10^5^	100	10^4^	[86]
	Au/starch/ITO	Carbon filament	10^3^	10^3^	10^4^	[110]
	Ag/pectin/FTO	Ag filament	450	100	500	[87]
	Ag/Ag‐doped CM:κ‐car/Pt	Ag filament	10^3^	50	10^4^	[86]
	Ag/ι‐car/Pt	Ag filament	10^7^	300	3 × 10^3^	[111]
	Al/glucose/Si	Interfacial modulation	10^3^	100	10^4^	[84]
	Ag/pectin/ITO	Ag filament	10^2^	500	10^4^	[96]
	Ag/CιC : Ag/ITO	Ag filament	10^2^	300	10^4^	[112]
	Mg/pectin/Mg	Mg filament	10^2^	500	10^−2^ to 10^6^	[67]
DNAs	Ag/DNA‐CTMA:NP/ITO	Ag filament	233	/	10^5^	[113]
	Au/(DNA)_10_/Au	Charge trap/detrap	30	100	10^6^	[90]
	Ag/DNA‐CTMA/ITO	Ag filament	10^2^	200	10^6^	[107]
	Pt/Cu^2+^DNA/FTO	Cu filament	10^3^	120	10^4^	[89]
Plants	Au/lignin/ITO	Carbon filament	/	/	/	[63]
	Ag/banana peel/Ti	Metalic filament	20–60	160	4×10^4^	[91]
	Ag/leaves/Ti/PET	Metalic filament	50	100	10^3^	[93]
	Mo/anthocyanin/Mo	Ion exchange	28	100	/	[17]
	Ag/melanin/ITO	Ag filament	10^4^	50	10^4^	[114]

**Figure 4:**
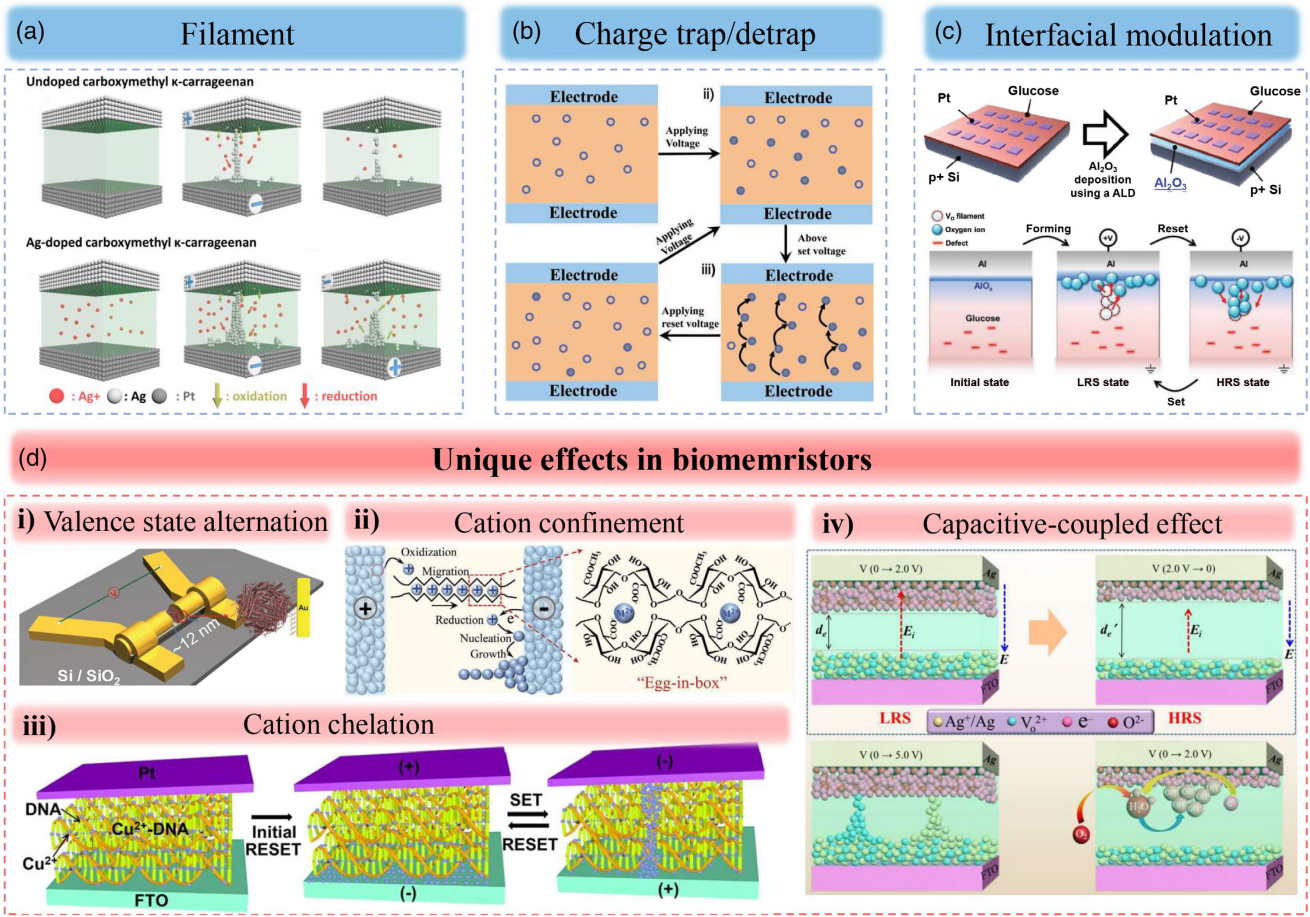
Switching mechanisms of biomemristors. a) Conductive filament. Reproduced with permission.^[^ ^86^ ^]^ Copyright 2018, American Chemical Society. b) Charge trap/detrap. Reproduced with permission.^[^ ^61^ ^]^ Copyright 2013, Wiley‐VCH. c) Interfacial modulation. Reproduced with permission.^[^ ^84^ ^]^ Copyright 2018, Wiley‐VCH. d) Unique effects of biomemristors such as i) valence state alternation. Reproduced with permission.^[^ ^58^ ^]^ Copyright 2011, Wiley‐VCH. ii) Cation confinement. Reproduced with permission.^[^ ^67^ ^]^ Copyright 2021, Wiley‐VCH. iii) Cation detaching/attaching. Reproduced with permission.^[^ ^89^ ^]^ Copyright 2019, IOP Publishing. iv) Capacitive‐coupled effect. Reproduced with permission.^[^ ^118^ ^]^ Copyright 2019, American Chemical Society.

In most oxide‐ and organic‐based memristors, mechanisms such as the formation of conductive filament, charge trapping/detrapping, or the modulation of interfacial barrier have been proposed.^[^ ^104^, ^115^, ^116^ ^]^ These physical mechanisms were also proposed to explain the switching behaviors of biomemristors (**Figure** **4**a–c). Detail descriptions of these mechanisms have already been presented in many reports.^[^ ^50^, ^51^, ^115^, ^116^, ^117^ ^]^ Herein, we would like to highlight some unique effects found in biomemristors (Figure 4d). For some biomaterials such as ferritin, pectin, DNA composites, and C_15_H_11_O_6_, unique effects such as valence state alternation, cation confinement, cation detaching/attaching, and capacitive‐coupled effect have been suggested. 1) Valence state alternation. Ferritin is a typical protein with a spherical shell and an active core of hydrous ferric oxide. In the core of hydrous ferric oxide, there are some disordered regions due to the existence of inorganic phosphates. These disorder regions can facilitate electron transport and determine the conductivity of ferritin. For iron, it is known that Fe(II) has more freedom than Fe(III). As proposed by Meng et al.,^[^ ^58^ ^]^ Fe(II) can be drifted more easily and thereby more disorder regions could be formed in the ferritin iron core. The valence state of iron atoms in the complex core can be alternated between Fe(II) and Fe(III) by the electrical‐induced redox reaction, which produces RS characteristics; 2) Cation confinement. In oxides and organics, the ion migration is random in nature. Xu et al.^[^ ^67^ ^]^ proposed that cations can be confined in acidic polysaccharides‐based memristor devices. Polysaccharides are long‐chain polymeric carbohydrates composed of monosaccharide units bound together by glycosidic linkages. Acidic polysaccharides are polysaccharides that contain carboxyl groups, phosphate groups, and/or sulfuric ester groups. In a typical acidic polysaccharide‐based memristor with active Mg as electrode, the Mg cations injected into the film can interact with ionizable acid. Therefore, the migration of cations can be guided and confined; 3) Cation detaching/attaching. DNA is the hereditary material in humans and almost all other organisms. The DNA molecule consists of two strands that wind around one another with a helix structure. Metal ions can stabilize the DNA structure by coordinating with the phosphodiester backbone. Abbas et al.^[^ ^89^ ^]^ reported a biomemristor with Cu^2+^‐doped salmon DNA as the switching layer. Unlike the conventional memristor devices in which cations come from the activate top electrode, Cu^2+^ can be detached and attached from the DNA duplex driven by the electric field. The device does not require the additional introduction of cations, which is critical for high endurance performance; and 4) Capacitive‐coupled effect. Sun et al.^[^ ^118^ ^]^ reported the observation of nonpinched current–voltage hysteresis characteristics in Ag/C_15_H_11_O_6_/FTO biomemristor. This extraordinary phenomenon can be attributed to the capacitive‐coupled effect. The drift and accumulation of positive charge and negative charge near different electrodes could reduce the effective dielectric thickness of the capacitor. The modulation of the effective dielectric thickness can change the device resistance. The work provided deep insight into the nonpinched switching behaviors of biomemristors.

## 3 Unique Characteristics of Biomemristors for Biorealistic Applications

In recent years, there has been growing interest in bioinspired neuromorphic systems using novel emerging devices. Memristors bear resemblance to a biological synapse as the device conductance (or resistance) can be incrementally modified by controlling charge or flux through it.^[^ ^27^ ^]^ Memristors can achieve various synaptic behaviors such as short‐term plasticity (STP), long‐term plasticity (LTP), spike‐timing‐dependent plasticity (STDP), paired‐pulse facilitation (PPF), and paired‐pulse depression (PPD). However, there is still a lack of memristor devices that bear resemblance to the structure, working mechanism, ionic modality, and operating voltage as biological synapses. Biomemristors offer opportunity for developing biosynapse‐like devices. Ion migration and redistribution have been extensively accepted as a main switching mechanism of memristor devices and the ionic dynamics of the device is the key to performance and functionality.^[^ ^119^, ^120^ ^]^ Despite the great achievements of qualitative synaptic functionality of memristors, there is still a lack in good control over internal ionic dynamics.^[^ ^121^, ^122^ ^]^ In addition, the operating voltages of the reported memristor devices are much higher than that of biological counterparts. Biomaterials can control the movement of ions through interaction with them. Biomaterials can also provide the platform to control the coupled conduction of protons and electrons and thus achieve synapse‐like behaviors by controlling proton activity. As a result, unique features such as faithful synaptic behaviors, biological bimodal switching, and biovoltage switching have been observed in biomemristors. Moreover, benefiting from the inherent biocompatibility of biomaterials and mechanical flexibility of the ultrathin device thickness, the biomemristor devices are suitable for human‐related applications, such as brain–machine interface, cognitive healthcare, and wearable and implantable neuromorphic electronics. These features make the biomemristor an ideal candidate for biorealistic neuromorphic applications.

### 3.1 Faithful Synaptic Behaviors

In biological synapses, ion channels are the basis for all of the electrical activities in the initiation, processing, and transmission of information. However, there is still a lack of memristor devices that bear resemblance to the structure, switching mechanism, and ionic transport modality as biological synapses. Therefore, it is desirable to develop biorealistic synaptic systems that are more reliable in terms of integration density, power efficiency, structure, and function.

**Figure 5:**
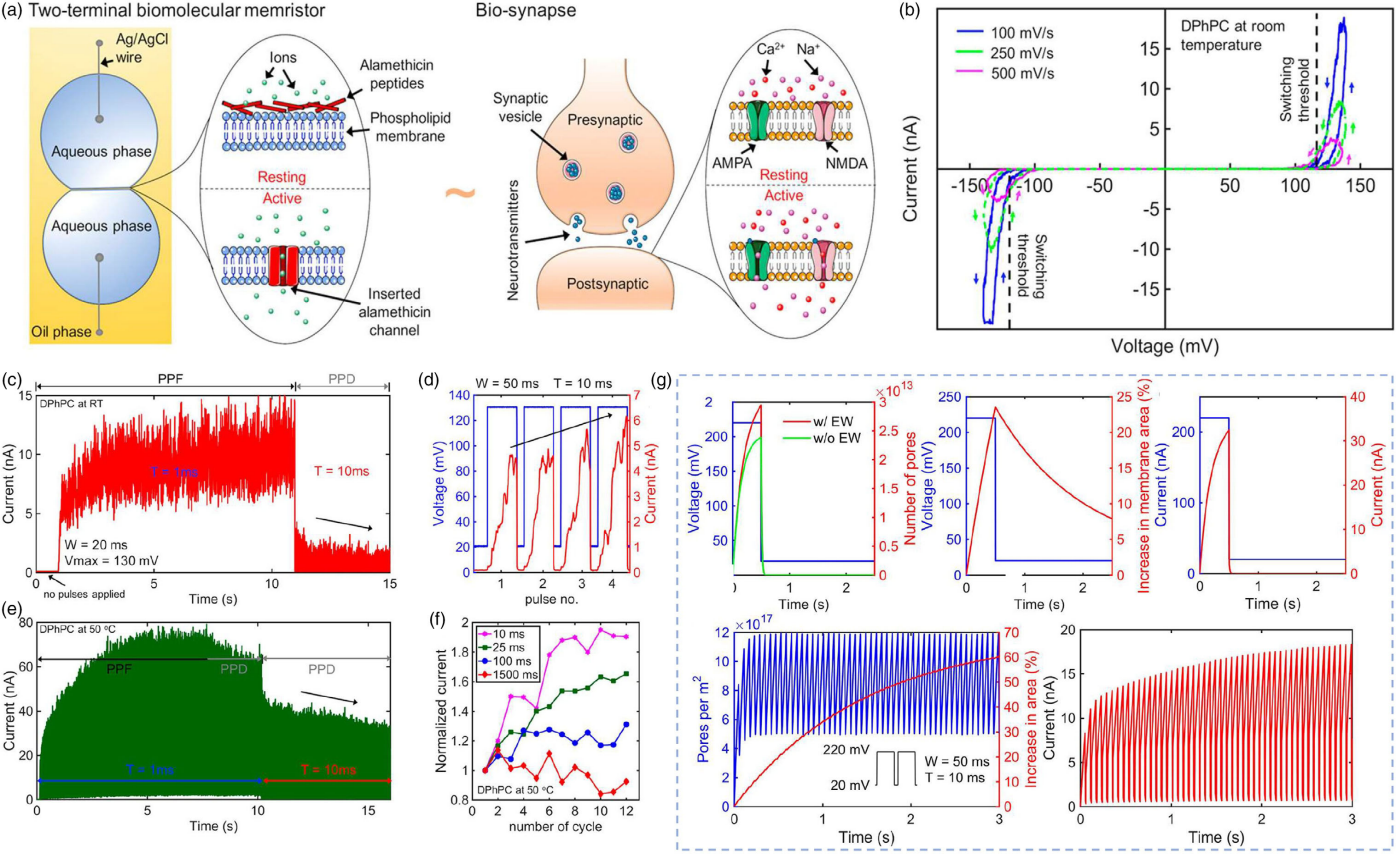
Memristor devices based on biomembranes. a) Schematic illustration of the biomemristive device (left) and the biosynapse (right). b) Switching behaviors of the device with different voltage sweep rates. c,e) PPF and PPD behaviors of the biomemristive device at room temperature and at 50 °C. d) PPF behavior emulated by pulses with width of 50 and interval of 10 ms. f) Normalized output current of the device triggered with pulses at various intervals. g) Simulated response of the biomemristor device. Reproduced with permission.^[^ ^64^ ^]^ Copyright 2018, American Chemical Society.

Sarles et al.^[^ ^64^, ^123^ ^]^ developed a biomemristor device with composition, structure, and mechanism similar to synapses. The device consisted of an ultrathin (3–5 nm) and highly insulating (≈10 GΩ) planar lipid bilayer assembled at the interface of aqueous droplets wrapped in lipid (**Figure** **5**a). Ion channels can be formed by the insertion of alamethicin (alm) peptides into the membrane. Above the threshold voltage, alm peptides can be inserted into the membrane to create ion transport channels. This feature of the biomemristor devices was analogous to those of ion channels in neurons (Figure 5b). The device exhibited bipolar threshold switching behavior with a low voltage of 120 mV. The switching characteristics were also dependent on the temperature and membrane composition. The voltage was increased and the current–voltage hysteresis was reduced by increasing the temperature to 50 °C. The voltage can also be lowered by replacing the diphytanoylphosphatidylcholine (DPhPC) lipids with porcine brain total lipid extracts. Short‐term synaptic plasticity, PPF, and PPD were emulated by pulses with different widths and intervals. Pulse (130 mV, 20 ms) with intervals of 1 ms for the first 10 s and 10 ms during subsequent pulses was applied on the device. As shown in Figure 5c, with the pulses at intervals of 1 ms, the device current increased until reaching a steady state, emulating PPF. During subsequent pulses at intervals of 10 ms, the current gradually reduced, emulating PPD. PPF also can be emulated by increasing the pulse width to 50 ms at intervals of 10 ms (Figure 5d). Interestingly, the PPF measured at 50 °C with varying pulse intervals showed a nonmonotonic rise in current (Figure 5e,f). This behavior was analogous to facilitation then depression due to excessive stimulation relevant to habituation in human sensory systems.^[^ ^42^ ^]^ When voltage was applied, the device conductance was highly dependent on two factors: 1) the insertion and aggregation of alm peptides to form conductive pores; and 2) the space for alm insertion. Simulation results indicated that pore insertion and relaxation are much faster than area changes (Figure 5g). When the voltage was larger than the threshold value, the device current was fully dependent on inserted channels. On the other hand, the area growth was more sensitive to the time intervals between each pulse. Therefore, PPF takes place when the pulse amplitude exceeds the threshold value with the time interval shorter than the relaxation time of the membrane area. In contrast, PPD can take place at the same pulse amplitude with larger time intervals. In addition, compared with solid‐state memristor devices, the biomemristor devices with biomolecules have advantages of power consumption (≈0.1–10 nW), and they were easily scalable via droplet‐based printing or microfluidic methods.

In human biological systems, the sensory nervous system is the cornerstone that builds up the connection between the external environment and organisms. Habituation plays an important role for the organism to adapt to desensitize and filter out irrelevantly repetitive information to be effective in assessing the novelty and importance of signals.^[^ ^45^ ^]^ The biomemristor devices with biomembranes offer the possibility of autonomic signal processing in visual, haptic, and olfactory sensors for neuromorphic hardware implementation.

### 3.2 Biological Bimodal Switching Behavior

The multimodal activation process is an important characteristic in biological systems. In neuronal synapses, chemicals cooperate in the process of electrical signal transfer that is determined by sensory and previous memory.^[^ ^124^ ^]^ Despite the great achievement in mimicking synaptic behavior, there is still a lack of the application of concerted behaviors to artificial sensory synapses. Protons have been investigated as mediators together with other neurotransmitters in synapses, which provide a platform for designing bioinspired devices.

**Figure 6:**
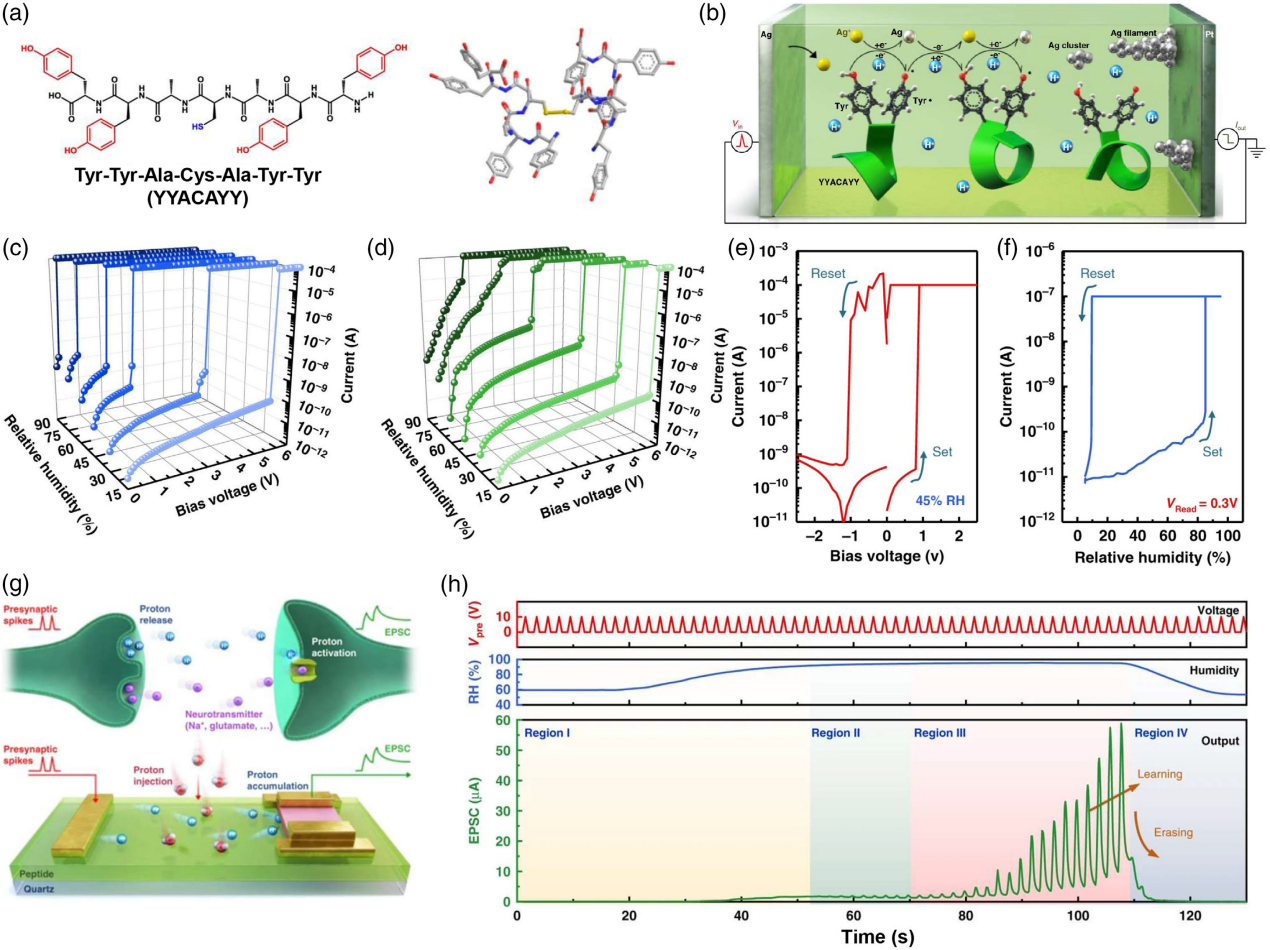
Bimodal switching of peptide‐based memristors. a) Chemical structure of the Y7C peptide. b) Schematic illustration of the switching mechanism of the Y7C film. c,d) Typical switching curves of the device under various relative humidities of H_2_O and D_2_O. e,f) Typical switching curves of the device with the voltage trigger mode (relative humidity: 45%) and humidity trigger mode (constant read voltage: 0.3 V). g) Schematic illustrations of the proton‐activated process in biological and artificial synapses. h) Humidity‐dependent plasticity of the artificial synapses. Reproduced under the terms of the CC‐BY 4.0 license.^[^ ^66^ ^]^ Copyright 2020, The Authors, published by Springer Nature.

In previous reports,^[^ ^125^, ^126^ ^]^ proton‐involved processes have been accepted as main mechanisms for some synaptic devices. Kwon et al.^[^ ^66^ ^]^ demonstrated a multimodal proton‐activated biomemristive device with tyrosine‐rich peptide films (**Figure** **6**a, Tyr–Tyr–Ala–Cys–la‐Tyr–Tyr (YYACAYY, Y7C)). There were two important characteristics of the Y7C film for bimodal operations of the device. 1) The Y7C film has high proton conductivity and the conductivity can be greatly enhanced by increasing the relative humidity; and 2) The migration and reduction of Ag ions can be promoted by both protons and the electric field.^[^ ^127^ ^]^ Therefore, the switching behaviors of the device can be triggered by both voltage and humidity with the help of proton‐mediated ion transport. The device working mechanism is similar to the conventional electrochemical metallization cells. Under voltage bias, Ag electrode is oxidized and dissolved into the peptide layer, and finally forms Ag filament, connecting top and bottom electrodes. (Figure 6b). It is noted that the reduction of Ag ions is dominated by charge transfer from the phenolic hydroxyl group in tyrosine to Ag ions.^[^ ^105^ ^]^ Protons are involved in charge transfer between tyrosine and Ag atoms. In the tyrosine‐rich Y7C peptide, the phenolic hydroxyl group acts as a hopping site for proton. Thus, it can be assumed that electron transfer is coupled with protonation and deprotonation of tyrosine (Figure 6b). They proposed that increasing the humidity can enhance the proton conduction in the Y7C peptide. As a result, the reduction of Ag ions can be promoted. They studied the effect of proton conduction of the peptide on the switching characteristic of the device. As shown in Figure 6c, the set voltage (at which the device resistance changed abruptly) of the device decreased from 4.6 to 0.4 V when the relative humidity increased from 15% to 90%. Similar results were also observed by replacing H_2_O vapor with deuterium oxide vapor (Figure 6d). The experimental results further evidenced that protons can be involved in the switching of the device. More importantly, the switching behavior of the device can be directly triggered by relative humidity change. The device showed bipolar switching characteristics with an on/off ratio of 10^6^ at 45% relative humidity (Figure 6e). With the application of a constant read bias (0.3 V), by the injection of H_2_O humidified air to continually increase the humidity, the device current abruptly increased when the relative humidity reached ≈90% (Figure 6f). The resistance states can be retained for over 10^4^ s regardless of whether it is switched by voltage bias or by humidity. The proton‐involved characteristic of the Y7C peptide film can also be used to develop synaptic transistors with mobile protons as the neurotransmitters (Figure 6g). The device had an in‐plane gate configuration and In–Ga–Zn–O (IGZO) was selected as channel material. The gate and IGZO channel were selected as presynaptic terminal and postsynaptic neurons, respectively. The humidity‐dependent plasticity was studied with a pulse width of 1 s, sequentially applied and relative humidity varied (Figure 6h). The plasticity can be divided into four regions depending on relative humidity. The postsynaptic current was not obvious at low humidity (Region I) and started to respond when RH exceeded 90% (Region II). At high humidity above 94%, the current can be continuously enhanced as a pulse was applied (Region III). When the humidity decreased, the postsynaptic current was suppressed quickly. The results highlighted the importance of proton in the operation of peptide‐based synaptic devices and advanced the synaptic application of peptides.

### 3.3 Biovoltage Operation

Memristor devices are promising candidates for neuromorphic applications. However, the operation voltages of memristor devices are generally larger than 0.5 V, which is much higher than the amplitude of biosystems (<100 mV).^[^ ^128^ ^]^ The mismatch of driving amplitude between the memristor devices and biological central neurons could limit the construction of memristor‐based neuromorphic systems and their functional versatility. In memristor devices based on electrochemical metallization, some biomaterials can facilitate redox reactions by donating electrons, which can enable the memristor operating with biovoltage.

**Figure 7:**
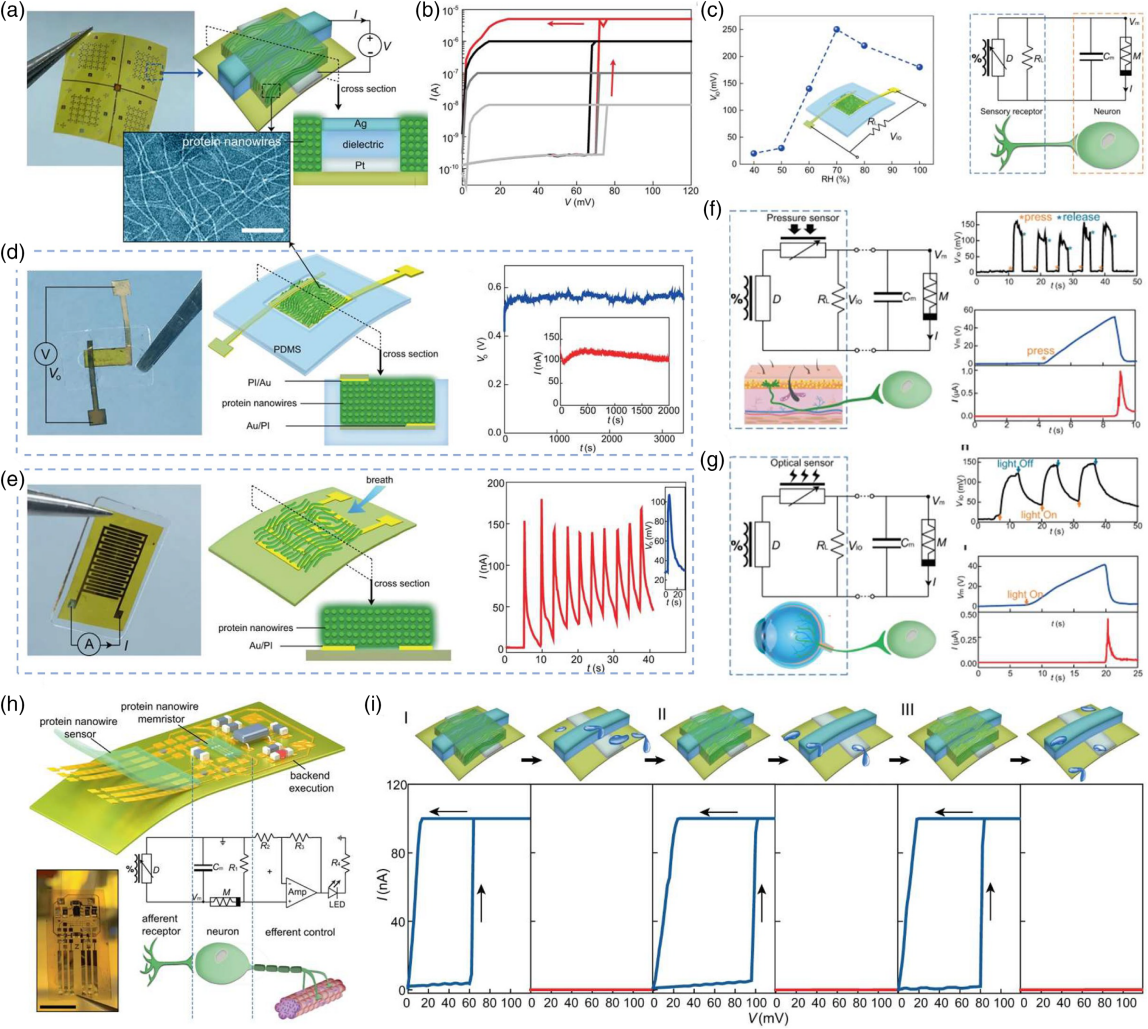
Biomemristor device with biovoltage operation for neuromorphic interfaces. a) The biomemristor arrays on a polyimide substrate. b) Typical *I–V* curves of the biomemristor with different current compliances. c) Protein nanowires‐based RH humidity sensory receptor with artificial neuron. d,e) Vertical protein and planar energy device with protein nanowire. f) Tactile neural interface. g) Optical neural interface. h) Schematic and circuit diagram of the wearable component for the neuromorphic interface. i) Schematics of reconfigurable functions of the biomemristor (top) and corresponding switching characteristics of the device at each stage (bottom). Reproduced under the terms of the CC‐BY 4.0 license.^[^ ^68^ ^]^ Copyright 2021, The Authors, published by Springer Nature.

Generally, the RS of an electrochemical‐type memristor often involves three steps: 1) anodic oxidization (M→M^+^ + e^−^); 2) M^+^ migration; and 3) cathodic reduction (M^+^ + e^−^ →M).^[^ ^129^, ^130^, ^131^ ^]^ Yao et al.^[^ ^83^ ^]^ proposed that cathodic reduction was an important factor determining the SET voltage. In this case, a catalyst in the memristor material that can reduce the reduction overpotential would be desirable for reducing the SET voltage of the device. Protein nanowires of the bacterium *G. sulfurreducens* have the ability to facilitate metal reduction with electrons derived from cellular metabolism.^[^ ^132^ ^]^ As hypothesized, Yao et al.^[^ ^83^ ^]^ developed an electrochemical‐type memristor device with Ag electrode and protein nanowires harvested from *G. sulfurreducens* as an active layer. Two types of devices with planar and vertical configurations were fabricated and the switching voltages of these two type devices were 45–80 and 40–80 mV, respectively. The ultralow switching voltage can address the inherent amplitude mismatch of neuromorphic devices between the sensing and computing signals. As some physiological signals are limited in amplitude, the device enables the construction of neuromorphic interfaces to directly process sensing signal sensors. In addition, protein nanowires harvested from *G. sulfurreducens* can generate electric power in the ambient environment and behave as a sensing component in humidity sensors. These features advance protein nanowires as building blocks for developing integrated neuromorphic interfaces. Yao et al.^[^ ^68^ ^]^ further developed a biomemristor device with a vertical structure consisting of a SiO_2_ layer sandwiched between Ag and Pt electrodes. The whole device was bedded in the protein nanowire film with a thickness of ≈500 nm (**Figure** **7**a). The memristor devices featured a switching voltage of 70 ± 3 mV at different compliance currents (Figure 7b). Two types of energy devices with vertical configurations and planar configurations were also fabricated. The vertical energy device was constructed with protein nanowires sandwiched between Au electrodes, which were further embedded between two polydimethylsiloxane layers (left of Figure 7d). The vertical moisture gradient can be built up in the ambient environment and sustained electric outputs can be observed (right of Figure 7d). By connecting the vertical energy device to a resistor, the output voltage can be modulated from below 30 to above 100 mV by changing the relative humidity (left of Figure 7c). The output voltage showed small fluctuations by further increasing the relative humidity, which could result from the change of the internal resistance of the protein nanowires. The planar energy device was fabricated with the protein nanowire film's (≈1 μm thick) deposition on a pair of interdigitated electrodes (left of Figure 7e). The device can generate an instant electric spike with breathing‐induced non‐uniform moisture adsorption (Right of Figure 7e). An artificial afferent circuit can be made with the connection of the vertical energy device, the biomemristor device, and a capacitor (Right of Figure 7c). The capacitor was used to emulate the membrane capacitance in a biological cell. At low relative humidity below 50%, the energy device had a low output voltage and the memristor device remained in a high resistance state. At high relative humidity above 70%, the high output voltage can charge the capacitor to increase the voltage dropped on the biomemristor device. The biomemristor device will be switched to a low resistance state and produce a current spike mimicking the action potential in neuronal firing. The artificial afferent circuit can also serve as a sensory interface responding to the stimulus of pressure and optics with the combination of the pressure sensor and optical sensor (Figure 7f,g). The resistance change of the sensors can be converted to voltage signals across the load resistor, leading to subsequent neuronal firing. They further developed a skin‐wearable integration with respiration as the stimulus (Figure 7h). This neuromorphic interface is very similar to that of biology, where sensing and decision can be accomplished without an external power source. A fully self‐sustaining neuromorphic interface is achieved based on the combination between biological amplitude signal processing and environmental energy harvesting. The devices also enable reconfigurable functions for adaptive microsystems (Figure 7i). The construction of neuromorphic interfaces with low operation voltage was highly desirable to facilitate direct data to realize data communication in biomachine interfaces.^[^ ^133^, ^134^ ^]^ The unique features of protein nanowire‐based biomemristor devices promote biomachine interfaces a step closer to biological integration.

### 3.4 Biological Conformability

High biological conformability of artificial synaptic devices is crucial for realizing human‐related applications, such as brain–machine interface, cognitive healthcare, wearable, and implantable neuromorphic electronics. To this end, the fabricated artificial synaptic devices are required not only to be sufficiently soft but also to be nontoxic, which ensures a seamless integration with human tissues or organs without causing an inflammatory response.

**Figure 8:**
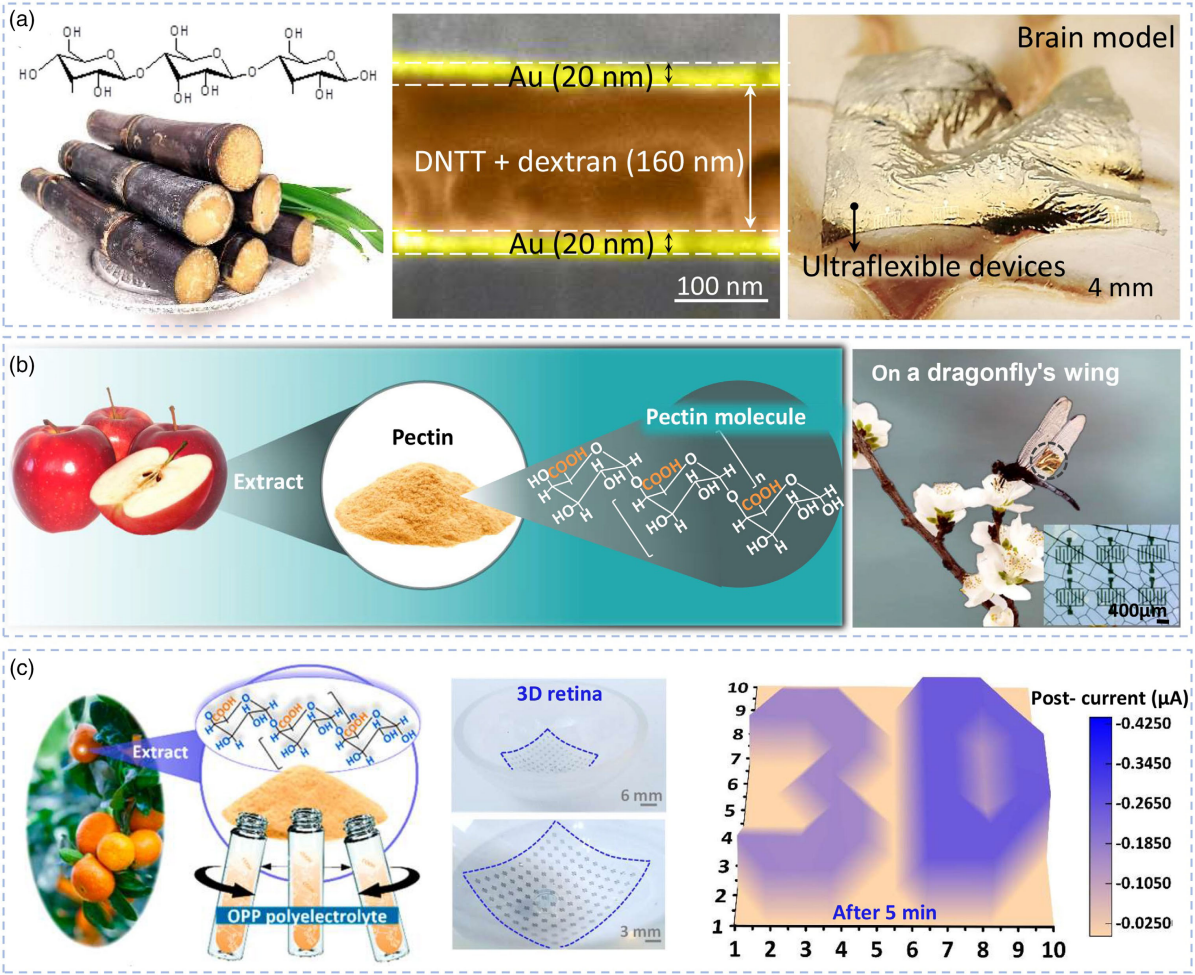
Ultraflexible artificial synaptic devices based on biomaterials. a) Dextran‐based ultraflexible organic synaptic transistors with the thickness of 200 nm. Left image: Reproduced with permission.^[^ ^135^ ^]^ Copyright 2020, Wiley‐VCH. Middle and right images: Reproduced with permission.^[^ ^136^ ^]^ Copyright 2020, Royal Society of Chemistry. b) Apple pectin‐based ultraflexible organic synaptic transistors with the thickness of 644 nm. Reproduced with permission.^[^ ^137^ ^]^ Copyright 2022, American Chemical Society. c) Orange peel pectin‐based ultraflexible organic synaptic transistors with the thickness of 475 nm for high‐sensitive hemispherical neuromorphic imaging systems. Reproduced with permission.^[^ ^138^ ^]^ Copyright 2022, Elsevier Ltd.

Ultraflexible artificial synaptic devices based on biomaterials are ideal candidates to achieve this application goal, due to their outstanding advantages of inherent biocompatibility of biomaterials and mechanical flexibility by the ultrathin device thickness. For instance, Yang et al.^[^ ^135^, ^136^ ^]^ reported dextran‐based ultraflexible, degradable organic synaptic transistors with thickness of only 200 nm (**Figure** **8**a). The resultant devices with outstanding mechanical flexibility could intimately conform to arbitrary‐shaped objects and at the same time maintain various synaptic plasticity. More importantly, they first explored the proton conduction behavior in the dextran membrane and found that protons mainly came from the self‐dissociation of water molecules and transferred by hydrogen bond network. In addition, the device has transient characteristics. They could degrade rapidly in the water environment without any toxic or harmful byproducts. Li et al.^[^ ^137^ ^]^ developed a natural biomaterial, apple pectin, as the dielectric layer and realized ultrasensitive and ultraflexible organic synaptic transistors with the thickness of 644 nm (Figure 8b). The lowest operating voltage for a single synaptic event is only −20 mV, which is fivefold (−100 mV) lower than the biological synaptic action potential. The obtained devices exhibited a sensitivity as high as 6.7 dB. At a low pulse voltage (−100 mV), the devices could simulate a wide variety of synaptic plasticity behaviors, such as short‐term‐to‐long‐term memory transition, paired pulse promotion, and the learning and forgetting process of the brain. Zhang et al.^[^ ^138^ ^]^ utilized a natural acidic polyelectrolyte as the dielectric layer and fabricated ultraflexible photoelectric synaptic transistors with the thickness of 475 nm (Figure 8c). The devices like biological retina could fit seamlessly to the 3D concave hemispherical surface and meanwhile possess high‐sensitive neuromorphic imaging function. It is worth mentioning that they found that the high‐proton‐concentration dielectric layer is crucial to the enhancement of the photosensitivity of photoelectric synaptic transistors. Compared with the low‐proton‐concentration dielectric layer (poly(vinylalcohol)), the photosensitivity of the acidic pectin‐based devices was improved by two orders of magnitude. Under a dim light condition (56 μW cm^−2^), the device array could simulate different behaviors of the human visual system, such as 3D image sensing and preprocessing, as well as image enhancement and erasure. These interesting works indicate that these ultraflexible and degradable devices have excellent biological conformability, presenting a remarkable advance toward developing natural biomaterials in biorealistic neuromorphic applications.

## 4 Conclusion and Outlook

The development of electronic devices with biomaterials is a hot research topic for environment and source concerns. This review has summarized the recent progress of natural biomaterial‐based memristor devices with biologically realistic functions, focusing on device configuration, memristor characteristics, working mechanisms, and functionality. The introduction of biomaterials broadens the functionalities of memristors, including environmental adaptability, biocompatibility, biodegradability, and so forth, which contribute to the development of “green” and “sustainable” electronics. Importantly, the biomemristor devices exhibit unique features for biorealistic neuromorphic applications. These studies build the foundation for future biomemristor devices that are capable of various neuromorphic applications.

Despite the advancements, the development of biomemristor devices is still at a preliminary stage and there are several materials and technical challenges that have to be addressed. Some challenges are highlighted: 1) Enhancing long‐term service performance of biomemristor devices. As the internal structure of biomaterials is sensitive to environmental humidity or temperatures, the long‐term working stability of the device should be improved.^[^ ^139^, ^140^, ^141^ ^]^ Encapsulation of the device would be an effective way to enhance the stability of biomemristor devices.^[^ ^142^ ^]^ For biomaterial, hydrophobic modification is an effective method to improve the resistance to moisture. Methods such as chemical grafting, plasma treatment, and polymerization have been reported to modify the hydrophobic properties of biomaterials.^[^ ^143^ ^]^ Molecular design and synthesis of thermally stable biomaterials are also required to improve the high‐temperature stability of devices.^[^ ^144^ ^]^ On the other hand, for memristor devices, the introduction of a thin buffer layer has been reported as an effective way to improve the switching performance.^[^ ^145^ ^]^ The protection of switching materials from air exposure also can enhance the long‐term environmental stability of memristors.^[^ ^146^ ^]^ Therefore, the introduction of the thin buffer layer is also expected to improve the environmental stability of biomemristors; 2) The exploration of unique features of biomemristor materials. The selection of suitable biomaterials used for memristor devices is still limited. Future efforts are still needed to study unique features of various biomaterials suitable for memristor applications; 3) Development of conductive biomaterials to replace the metal electrode. It is difficult to say that the biomemristor is biocompatible when using metal or ITO as the electrodes. Electrode is essential for memristor devices. Ag and Mg are often used for electrochemical and dissoluble purposes. After the dissolution of the biomemristor devices, the metal will remain in the environment.^[^ ^147^, ^148^, ^149^ ^]^ In addition, flexibility is one of the important features of biomaterials‐based electronic devices for wearable applications.^[^ ^150^, ^151^, ^152^ ^]^ However, the rigid nature of metals restricts their wearable applications. Future efforts in developing conductive biomaterials as electrodes are necessary to achieve intrinsically biocompatible, biodegradable, and stretchable devices; 4) Future advances in techniques for high‐density integration of biomemristor devices. Many biomaterials are not compatible with traditional photolithography. The integrity of materials could be destroyed by organic solvents or ultraviolet light in the traditional photolithography process. Novel fabrication and integration technologies are still needed to overcome this challenge. Photolithography‐compatible fabrication methods, where each functional layer is prepared discretely on the rigid hydrophobic substrate and striped and stacked together to form a whole device, are expected to solve the integration difficulties of biomemristors^[^ ^153^, ^154^ ^]^; 5) Further improvement of the switching performance of biomemristor devices. Though outstanding performances have been reported in some biomemristor devices such as low switching voltage, fast switching speed, high switching uniformity, and low power consumption, it is still a challenge to combine all the superior characteristics in a single device. Further works from the perspective of material and device optimization are needed to improve the switching performance of the device. Precise control of the ionic procedure could be the most prominent requirements for improving RS performance. For some biomaterials, the existence of functional groups offers the possibility for ion regulation. It has been reported that for biomaterials with acidic and/or hydroxy groups, cations can interact with these groups.^[^ ^66^, ^85^ ^]^ Therefore, modification of these groups would be an effective solution for improving the resistance switching performance. In addition, strategies previously proposed for improving the switching performance of memristors (such as embedding tip electrode, inserting buffer layer) would also have some applicability for biomaterials; 6) Improve the water resistance of the biomaterials. So far, there is no experimental demonstration on the implantation of biomemristors into biological systems. One challenge could be the water‐dissolvable characteristics of biomaterials. For example, body fluid in humans may damage the device. The hydrophobic modification of biomaterials would offer an effective way to improve their resistance to water. Methods such as chemical grafting, plasma treatment, and polymerization have been reported to modify the hydrophobic properties of biomaterials.^[^ ^143^, ^155^ ^]^ Therefore, with the development of surface modification technology of biomaterials, it is expected that biomemristors could be implantated into biological systems. Research effort and progress are still required.

Though biomemristor devices have advantages in terms of biodegradability and biocompatibility, they should not be regarded as the total replacement for inorganic counterparts from the perspectives of working stability, device integration, and switching performance. At present, biomemristor devices can be regarded as an extension of memristor devices with specific and novel applications by virtue of some unique biorelastic characteristics. It is believed that the biomemristor device can play an important role in developing neuromorphic electronic systems.

## Conflict of Interest

The authors declare no conflict of interest.
